# Editorial: Edema in heart failure with reduced ejection fraction

**DOI:** 10.3389/fcvm.2023.1141937

**Published:** 2023-02-07

**Authors:** Inna P. Gladysheva, Ryan D. Sullivan, Pierpaolo Pellicori

**Affiliations:** ^1^Department of Internal Medicine and Translational Cardiovascular Research Center, University of Arizona College of Medicine – Phoenix, Phoenix, AZ, United States; ^2^College of Medical, Veterinary and Life Sciences, School of Cardiovascular and Metabolic Health, University of Glasgow, Glasgow, United Kingdom

**Keywords:** edema, heart failure, congestion, cardiac dysfunction, biomarkers

Epidemiological data from Europe and North America suggest that 1–1.5% of the adult population has heart failure with reduced left ventricular ejection fraction (HFrEF). Development of edema (pulmonary or systemic) is a major hallmark of heart failure (HF) and a key driver of symptoms, such as exertional dyspnea and fatigue, reduced quality of life, morbidity and mortality (1).

Timely identification and treatment of edema are critical strategies in HF management, but are both difficult, for many reasons. A certain degree of peripheral edema is frequent in the elderly, but it might be due to poor mobility or use of treatments such as dihydropyridine calcium channel blockers, rather than HF. Exertional dyspnea is often attributed to old age or can be caused by a long list of highly prevalent conditions, including chronic lung disease, obesity and anemia, that frequently overlap and might confound a HF diagnosis. Therefore, a diagnosis of HFrEF is usually made late, when patients are already admitted to the hospital; by then, high doses of diuretics are required to improve symptoms and peripheral or pulmonary edema. Unfortunately, the use of decongestive therapies is still highly subjective and not guided by strong evidence (2).

The research and review articles contributing to this Research Topic focus on the pathophysiological mechanisms and molecular pathways underlying edema development, discuss potential therapeutic targets (beyond decongestive therapy with diuretics) and evaluate tools – biomarkers, imaging modalities, and algorithms that might facilitate diagnosis and monitoring of edema or, in other words, the management of HF.

The pathophysiology of edema formation during the advanced stages of HF is multifactorial and can be influenced by concurrent comorbidities, in particular renal failure. In patients with HFrEF, edema is attributed to the pathological extracellular fluid accumulation in the interstitial space resulting from renal salt and water retention and impaired extra fluid removal mechanisms (including the lymphatic system) from the interstitial to intravascular space. Persistent overactivation of the renin-angiotensin-aldosterone system, impairment of natriuretic peptide system and dysregulation of other hormonal axes concur to cause salt and water retention and worsen edema further (3). Effective decongestion and maintenance of euvolemia in patients with HFrEF are challenging, as therapeutic interventions may lead to intravascular volume depletion, hypotension, electrolyte abnormalities, and worsening renal function. In this Research Topic, Abassi et al. and Aronson comprehensively overviewed mechanisms underlying HF-associated edema formation and discussed novel strategies that might facilitate effective decongestion. The potential clinical relevance of novel biomarkers that reflect the activation of different pathways leading to edema and driving progression of HFrEF has been reviewed and discussed by Chiorescu et al..

Routinely collected, readily available diagnostic information can improve risk stratification for patients with dyspnea presenting to an emergency department. In another manuscripts published in this issue, Kobayashi et al. assessed the clinical value of combining a chest x-ray congestion score and plasma volume estimated from hemoglobin and hematocrit at admission in a cohort of 252 patients presenting to an emergency department with acute dyspnea, who were subsequently diagnosed with acute HF. They found that patients with both evidence of pulmonary and intravascular edema had a greater risk of dying in hospital compared to those who had either of the two features, or none.

To extend the clinical algorithm that characterizes the hemodynamic status and type of congestion in patients with acute HF, Palazzuoli et al. proposed incorporating a bedside echocardiographic examination into the Stevenson classification. The comparative analysis of Stevenson classification data and echocardiographic findings reported by this research team supports the benefits of echocardiographic evaluation for improving characterization of the HF patient's clinical profile and diagnosis.

Intravascular volume status can be estimated using ultrasound by measuring the inferior vena cava (IVC) or internal jugular vein (iJV) diameter and their responses to simple respiratory maneuvers (4). A dilated and stiff IVC or iJV on ultrasound identifies patients with more severe congestion and a greater risk of HF hospitalization or premature death. Automated algorithms that track displacement of vessels walls in real time are currently under-development, to quantify changes in vessel size with more precision. In this issue, Mesin et al. outline current development in this field and highlight the clinical potential of these novel, non-invasive, diagnostic methods.

Implementation of modern treatments, for instance sacubitril/valsartan and sodium-glucose co-transporter-2 inhibitors (5, 6), in clinical practice is required to improve management of congestion and, importantly, long term outcomes in patients with HFrEF. However, further research is needed to develop affordable methods and strategies that combine biomarkers, imaging modalities and other diagnostic tools to detect, quantify and monitor edema due to cardiac dysfunction timely and with precision (Figure 1). Lastly, but not of less importance, future trials should determine how to combine different classes of diuretics to treat severe edema in patients with HFrEF, and who can be safely withdrawn from diuretic therapy. Personalized management of edema is an important clinical problem and remains a hot Research Topic.

**Figure 1:**
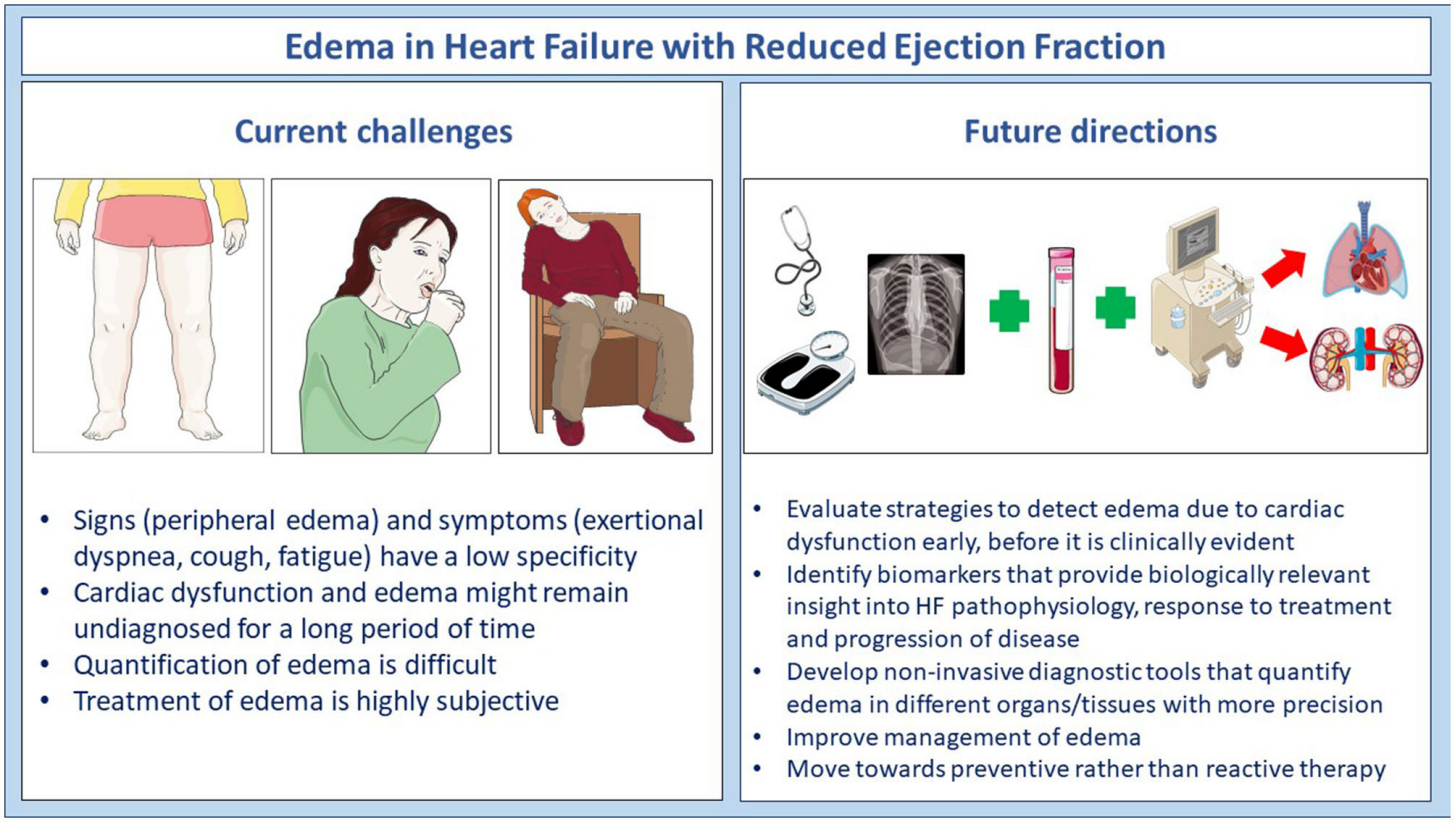
Current challenges and future directions in the identification and management of edema in patients with heart failure.

## Author contributions

All authors listed have made a substantial, direct, and intellectual contribution to the work and approved it for publication.
